# Extraction and Analysis of Dynamic Functional Connectome Patterns in Migraine Sufferers: A Resting-State fMRI Study

**DOI:** 10.1155/2021/6614520

**Published:** 2021-04-17

**Authors:** Weifang Nie, Weiming Zeng, Jiajun Yang, Yuhu Shi, Le Zhao, Ying Li, Dunyao Chen, Jin Deng, Nizhuan Wang

**Affiliations:** ^1^Lab of Digital Image and Intelligent Computation, Shanghai Maritime University, Shanghai 201306, China; ^2^Department of Neurology, Shanghai Jiao Tong University Affiliated Sixth People's Hospital, Shanghai 201306, China; ^3^Artificial Intelligence and Neuro-Informatics Engineering (ARINE) Laboratory, School of Computer Engineering, Jiangsu Ocean University, Lianyungang 222002, China

## Abstract

Migraine seriously affects the physical and mental health of patients because of its recurrence and the hypersensitivity to the environment that it causes. However, the pathogenesis and pathophysiology of migraine are not fully understood. We addressed this issue in the present study using an autodynamic functional connectome model (A-DFCM) with twice-clustering to compare dynamic functional connectome patterns (DFCPs) from resting-state functional magnetic resonance imaging data from migraine patients and normal control subjects. We used automatic localization of segment points to improve the efficiency of the model, and intergroup differences and network metrics were analyzed to identify the neural mechanisms of migraine. Using the A-DFCM model, we identified 17 DFCPs—including 1 that was specific and 16 that were general—based on intergroup differences. The specific DFCP was closely associated with neuronal dysfunction in migraine, whereas the general DFCPs showed that the 2 groups had similar functional topology as well as differences in the brain resting state. An analysis of network metrics revealed the critical brain regions in the specific DFCP; these were not only distributed in brain areas related to pain such as Brodmann area 1/2/3, basal ganglia, and thalamus but also located in regions that have been implicated in migraine symptoms such as the occipital lobe. An analysis of the dissimilarities in general DFCPs between the 2 groups identified 6 brain areas belonging to the so-called pain matrix. Our findings provide insight into the neural mechanisms of migraine while also identifying neuroimaging biomarkers that can aid in the diagnosis or monitoring of migraine patients.

## 1. Introduction

Migraine is a headache disorder characterized by pulsating recurrent pain attacks combined with nausea, vomiting, sleep disorder, and hypersensitivity to visual, auditory, olfactory, and somatosensory stimuli [1]. Migraine affects approximately 14.7% of the global population and has a hereditary component [2]; because of the high morbidity and difficulty in treatment, migraine can severely limit patients' work efficiency and quality of life. Although many theories have been proposed for the etiology of migraine (including the vasogenic, cortical spreading depression, and trigeminal vascular theories), the neurologic basis is not well understood.

Resting-state functional magnetic resonance imaging (rs-fMRI) technology is a noninvasive method for measuring the spontaneous activity of neurons [3] that has enabled the identification of several brain regions involved in the pathogenesis of migraine based on the amplitude of low-frequency fluctuations (ALFFs) in the resting state [4]. For example, ALFF abnormalities have been observed in multiple brain regions in migraine patients including the right insular lobe, prefrontal cortex (PFC), and medial (m)PFC [5]. Other studies have used regional homogeneity (ReHo) to analyze the synchronization of local activity in the brain [6] and have found altered ReHo values in multiple brain regions in migraine patients such as those related to pain [7–9]. The causes of migraine have also been explored by analyzing correlations in activity between a predefined seed region and other brain areas; it was found that functional connectivity (FC) between some seed points (e.g., cerebellum, insula, frontal lobe, cingulate gyrus, superior marginal gyrus, and brainstem) and other regions was increased or decreased in migraine, resulting in changes in the integration of pain information [10–12]. Additionally, independent component analysis (ICA), temporal clustering analysis, and small-world network theory [13–17] have been applied to the study of migraine and have revealed that long-term repetitive pain stimulation can lead to abnormal synergistic processing and topologic abnormalities in brain functional networks [18, 19].

Although the abovementioned studies have provided insight into the neurologic mechanisms of migraine, they involved the analysis of static rs-fMRI data. Meanwhile, the brain exhibits complex spatial and temporal variations during dynamic processing as well as functional activity alterations in the resting state [20, 21] that must be taken into account. Dynamic (d)FC analysis evaluates brain activity fluctuations on the scale of seconds to minutes [22, 23] and has been used to establish activity profiles of thalamocortical in migraine patients [24]. Combined with Group ICA, dFC in brain networks has also been examined to find the functional characteristics of the migraine brain and revealed more functional networks related to migraine than the conventional static analysis [25]. Based on comparing dynamic functional connectivity patterns in migraine versus persistent posttraumatic headache, significant differences between migraine and persistent posttraumatic headache for 10 region pairs were found [26]. From the perspective of multichannel hierarchy, dFC combined topology properties have been investigated between large-scale brain networks in migraine patients, and results showed that the dynamic FCs and corresponding global topology properties have significant differences between migraine patients and healthy controls, while local topological properties and dynamic fluctuations were easily affected by window widths [27].

The above findings suggest that migraine can be investigated in terms of functional dynamics. To this end, in this study, we used an auto- (A-) DFCM with twice-clustering to establish a dFC profile in migraine based on rs-fMRI data. We speculated that in addition to migraine-specific dFC patterns (DFCPs), there are general DFCPs that are also present in both subjects. The results showed that the specific pattern and the particular brain regions can be detected for migraine patients, which can be useful for the clinical diagnosis of migraine.

## 2. Materials and Methods

### 2.1. Data Acquisition

A total of 34 migraineurs (19 males and 15 females; average age: 36.12 years (range: 17–58 years)) were recruited at the Department of Neurology of Shanghai Sixth People's Hospital East Affiliated with Shanghai University of Medicine and Health Science. All procedures were approved by the Independent Ethics Committee of Shanghai Sixth People's Hospital East Campus, and all participants provided informed consent before participating in the study. Migraine patients were diagnosed with chronic migraine according to International Classification of Headache Disorders 3rd Edition (beta version) criteria [28]. rs-fMRI data were acquired using a 3 T scanner (Siemens, Erlangen, Germany). During the scan, subjects were awake and were instructed not to think and to remain still. The scanning parameters were as follows: slice number = 38 (covering all brain areas), repetition time (TR) = 3.0 s, number of time points = 160, scan resolution = 64 × 64, on-chip resolution = 4 × 4 mm, and slice thickness = 4 mm. rs-fMRI data of 34 normal control subjects (14 males and 20 females; average age: 21.4 years (range: 18-26 years)) for the control group were obtained from a free public database (http://fcon_1000.projects.nitrc.org/fcpClassic/FcpTable.html) and were abbreviated as Beijing_Zhang which were released by Dr. Yu-Feng Zang; the parameters for the scans were as follows: slice number = 33 (covering all brain areas), TR = 2.0 s, number of time points = 225, scan resolution = 64 × 64, on-chip resolution = 3.13 × 3.13 mm, and slice thickness = 3.6 mm. All of the above data had been used in a published paper [27]. To check the repeatability of the consequence, additional rs-fMRI data of 34 normal control subjects (19 males and 15 females; average age: 36.18 years (range: 19-62 years)) were also obtained from a free public database (http://fcon_1000.projects.nitrc.org/fcpClassic/FcpTable.html) and were abbreviated as ICBM which were released by Alan C. Evans; the parameters for the scans were as follows: slice number = 23 (covering all brain areas), TR = 2.0 s, and number of time points = 128. The *p* value for ages was 0.9844 based on the unpaired *t*-test with Welch's correction and indicated no significant difference between ICBM and migraineurs, which means the data from ICBM were gender-matched and age-matched with the migraineur group.

### 2.2. Data Preprocessing

rs-fMRI data were preprocessed with Data Processing Assistant for Resting-State fMRI [29], which involved slice timing and head motion correction, spatial normalization, and spatial smoothing. Before slice timing correction, the first 10 time points were removed, and the middle slice was used as the reference frame for the correction. Sinc interpolation and 6° transformation were applied to eliminate temporal and spatial offsets, respectively. To minimize artifacts, data for which there was >1.5 mm displacement in any direction or head rotation > 1.5° were discarded. Spatial normalization involved reslicing to 2 × 2 × 2 mm using an echoplanar imaging template released by the Montreal Neurological Institute. A Gaussian kernel of 6 mm was applied to smooth the data.

### 2.3. A-DFCM with Twice-Clustering

**Figure 1:**
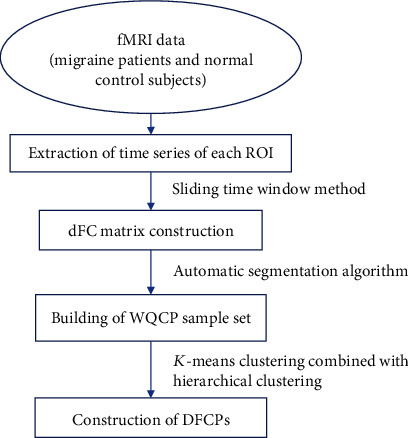
Framework of A-DFCM with twice-clustering.

The framework for A-DFCM with twice-clustering included dFC analysis using a sliding time window, an automatic segmentation algorithm, and *K*-means clustering combined with hierarchical clustering (Figure 1). The first step involved extracting the time series of each region of interest (ROI). Based on the Pearson correlation, dFC matrices were constructed with the sliding time window method. An automatic segmentation algorithm was used to construct the whole-brain quasistable connectome pattern (WQCP) sample set (Section 2.4). A twice-clustering algorithm that included *K*-means and hierarchical clustering was used to obtain cluster labels for each sample in a WQCP set. The specific and general DFCPs were identified by analyzing intergroup differences in the distribution ratio for each DFCP (Section 2.5). Local network metrics were calculated to identify brain regions that were important in the specific DFCP. Finally, significantly different dFCs were extracted with the independent 2-sample *t*-test for each general DFCP; intersecting dFCs were identified as those existing in all general DFPCs and were used to evaluate differences between the 2 groups (Section 2.6).

### 2.4. Automatic Generation of WQCPs

For each subject, the Brainnetome Atlas [30] with 246 ROIs in 210 cortical and 36 subcortical subregions was used to extract the time series comprising preprocessed rs-fMRI signal values. The mean time series was calculated based on the average rs-fMRI signal values of each ROI. For each subject, the mean time series was defined as

(1) $$\begin{array}{c} T_{n}=\left[t_{n,1},t_{n,2},\cdots ,t_{n,M}\right], \end{array}$$

where *t*_*n*,*i*_ (1 ≤ *n* ≤ 246; 1 ≤ *I* ≤ *M*) is the average time signal value of all voxels in the *n*th ROI at the *i*th time point and *M* is the length of time points.

As information processing in the brain changes over time, FC in the brain undergoes temporal transformation [20, 31]. A sliding time window [32] was used to evaluate FC dynamics. The mean time series *T*_*n*_ was first partitioned into temporal segments; the segment in the *n*th ROI at the *i*th time point was expressed as

(2) $$\begin{array}{c} {\text{ST}}_{n,i}=\left[t_{n,i},t_{n,i+1},\cdots ,t_{n,w+i+1}\right], \end{array}$$

where 1 ≤ *n* ≤ 246 and 1 ≤ *i* ≤ (*M* − *W* + 1); *W* is the window length; and step length is 1. We determined that *W* was 12 for migraine patients and 18 for normal control subjects, with a time duration of 36 s.

The Pearson correlation between 2 temporal segments at the same time point in all ROIs was calculated to determine dFC, as shown below:

(3) $$\begin{array}{c} {\text{Corr}}_{i,j,t}=\frac{\text{Cov}\left({\text{ST}}_{i,t},{\text{ST}}_{j,t}\right)}{\sqrt{D\left({\text{ST}}_{i,t}\right)}\;\sqrt{D\left({\text{ST}}_{j,t}\right)}},\text{if }i\neq j,\\ {\text{Corr}}_{i,j,t}=0,\text{if }i=j,\\ \text{d}{\text{FC}}_{i,j,t}=\left\{{\text{Corr}}_{i,j,t}\;|\;i,j\in \left[1,246\right];t\leq M-W+1\right\}. \end{array}$$

A dFC matrix was constructed with all dFC values at a given time point for the same subject. The dFC matrix at the *b*th time point was defined as

(4) $$\begin{array}{c} {\text{dFC}M}_{b}=\left[\begin{array}{cccccc} {\text{dFC}}_{1,1,b} & \text{d}{\text{FC}}_{1,2,b}\; & \cdots & \cdots & \cdots & {\text{dFC}}_{1,246,b}\\ \text{d}{\text{FC}}_{2,1,b} & {\text{dFC}}_{2,1,b} & \cdots & \cdots & \cdots & {\text{dFC}}_{2,246,b}\\ \vdots & \vdots & \vdots & \vdots & \vdots & \vdots \\ \text{d}{\text{FC}}_{u,1,b} & \text{d}{\text{FC}}_{u,1,b} & \cdots & {\text{dFC}}_{u,v,b} & \cdots & \text{d}{\text{FC}}_{u,246,b}\\ \vdots & \vdots & \vdots & \vdots & \vdots & \vdots \\ {\text{dFC}}_{246,1,b} & \text{d}{\text{FC}}_{246,2,b} & \cdots & \cdots & \cdots & \text{d}{\text{FC}}_{246,246,b} \end{array}\right], \end{array}$$

where 1 ≤ *b* ≤ (*M* − *W* + 1), 1 ≤ *u* ≤ 246, and 1 ≤ *v* ≤ 246.

The dFC strength (dFCS) vector was obtained by adding all absolute values of dFC for the same ROI, as follows:

(5) $$\begin{array}{c} {\text{dFCSV}}_{b}=\left[\begin{array}{c} \overset{246}{\underset{c=1}{\sum }}\text{a}\text{bs}\left({\text{dFC}}_{1,c,b}\right)\\ \overset{246}{\underset{c=1}{\sum }}\text{a}\text{bs}\left({\text{dFC}}_{2,c,b}\right)\\ \vdots \\ \overset{246}{\underset{c=1}{\sum }}\text{a}\text{bs}\left({\text{dFC}}_{i,c,b}\right)\\ \vdots \\ \overset{246}{\underset{c=1}{\sum }}\text{a}\text{bs}\left({\text{dFC}}_{246,c,b}\right) \end{array}\right], \end{array}$$

where 1 ≤ *b* ≤ (*M* − *W* + 1) and 1 ≤ *i* ≤ 246.

All dFCS vectors were ordered according to time point, and the dFCS matrix of each subject was constructed as shown below:

(6) $$\begin{array}{c} \text{dFCSM}=\left[{\text{dFCSV}}_{1},{\text{dFCSV}}_{2},\cdots ,{\text{dFCSV}}_{b},\cdots ,{\text{dFCSV}}_{M-W+1}\right]. \end{array}$$

**Figure 2:**
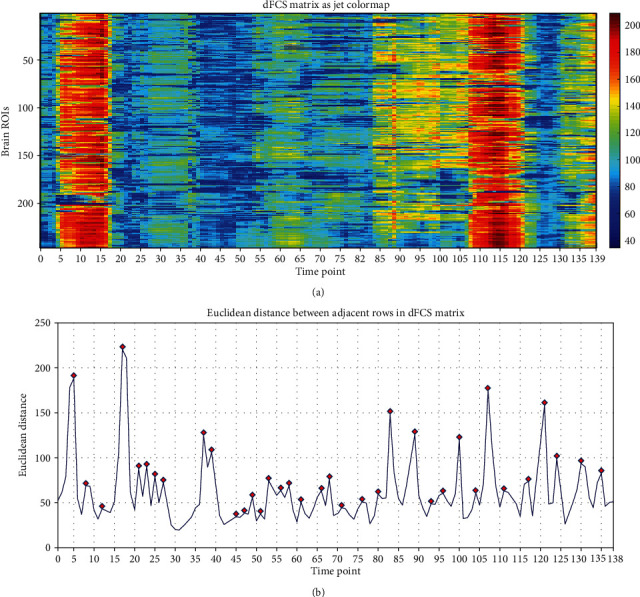
dFCS matrix shown as jet colormaps and curve of Euclidean distance values.

The illustrated dFCS matrix was divided into several segments based on similarities in the color of adjacent rows. In previous work [33, 34], segment points were manually located; in order to improve efficiency in the present study, automatic localization was adopted by calculating the Euclidean distance between adjacent rows. If the distance between the *i*th and (*i* + 1)th rows was larger than that between the *i*th and (*i* − 1)th rows and between the (*i* + 1)th and (*i* + 2)th rows, then the point *i* was selected as the segment point. A dFCS matrix is shown as a jet colormap in Figure 2 along with Euclidean distances between adjacent rows in the dFCS matrix (Figure 2); it can be seen that the segment points are the peak points in the Euclidean distance curve and separate the matrix into distinct components.

Once the segment points were located and marked, the dFCS matrix was divided into several sections; for each of these, the WQCP (246 × 1) vector was obtained by time averaging. WQCPs from all subjects were assembled to form the WQCP dataset, which was analyzed with a machine learning method to obtain brain DFCPs.

### 2.5. Identification of Specific/General DFCPs

Specific DFCPs describe the brain state specific to migraine patients, whereas general DFCPs show similarities and differences in the brain states of the 2 groups. In order to identify specific and general DFCPs, each WQCP sample in the WQCP dataset of the migraine and normal control groups was partitioned into different clusters by applying a twice-clustering algorithm that included *K*-means and hierarchical clustering. In traditional *K*-means clustering, initial cluster centers are randomly generated, which hinders the optimal performance of the algorithm and makes it inconvenient to use. Therefore, hierarchical clustering was used to determine the initial centers, and all WQCP samples were grouped into 17 clusters based on the Davies-Bouldin index [35]; these were considered the DFCPs that revealed dynamic functional layouts of the brain in the 2 states (i.e., migraine and normal). All dFC matrices matching each WQCP of each cluster were recaptured. Mean dFC matrices with a dimension of 246 × 246 were obtained by averaging all dFC matrices in the time dimension for migraine patients, normal control subjects, and both groups and were defined as the respective centroids.

After the grouping procedure, each WQCP sample had a DFCP label from 1 to 17. The distribution ratio of WQCP samples in each DFCP for each subject was calculated. We also compared the distribution ratio between migraine patients and normal control subjects with the independent 2-sample *t*-test for each DFCP using a threshold of *p* < (0.0001/17). The DFCP was deemed specific if it showed a significant difference in the distribution ratio between the 2 groups; otherwise, it was considered a general DFCP.

### 2.6. Analysis of Specific/General DFCPs

For the specific DFCP, local network metrics were calculated based on its centroid in the migraine group for extraction of critical brain regions. For each node, 2 local metrics—i.e., degree *k* [36] and participation coefficient *p* [37]—were calculated using the Brain Connectivity Toolbox (https://sites.google.com/site/bctnet/); these are described in Supplementary Table S1. Threshold values (i.e., ratio of the number of existing edges to the maximum possible number of edges in the graph [=30,135]) used in this study ranged from 10% to 40%. As there was a high correlation between metrics and threshold values for each node and no uniform criterion for threshold selection, the area under the curve (AUC) of each metric was calculated to eliminate threshold randomness [38].

Critical brain areas are often identified using degree *k* and participation coefficient *p* [39, 40]. The greater the degree of a node, the more edges are connected to that node; thus, the degree is the simplest and most common way of identifying key nodes. The participation coefficient is another index for evaluating the importance of nodes; a large coefficient indicates involvement in more modules and can be considered the center of information integration. In this study, we identified key brain areas based on *k* and *p* values >1 standard deviation above the mean for all nodes in the network [41].

For general DFCPs, intergroup differences were evaluated by comparing dFCs in the dFC matrices with the independent sample *t*-test at a threshold *p* value <(0.0001/30135). The number of dynamic functional connections in 1 dFC matrix was 30,135. Brain regions corresponding to the detected dynamic functional connections were identified.

## 3. Results

### 3.1. Ratio Distribution of WQCP Samples under Resampling

**Figure 3:**
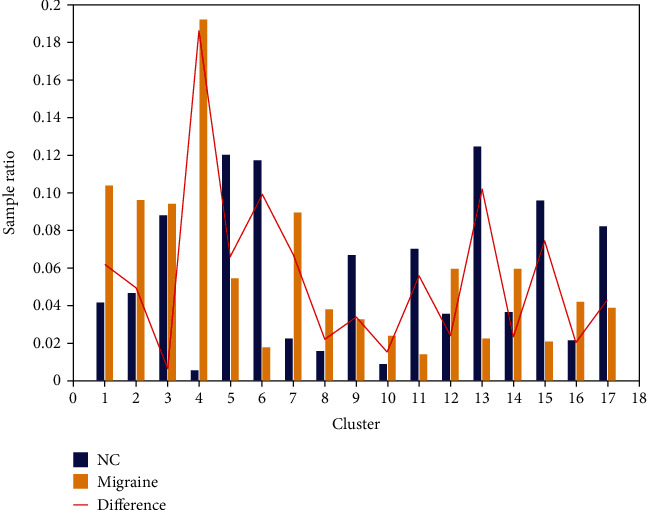
Ratio distributions of WQCP samples for migraine patients and normal control (NC) subjects. Orange and blue bars represent migraine and normal control groups, respectively; the red curve shows the difference in ratio distributions of the 2 groups.

A total of 17 DFCPs were extracted using the twice-clustering algorithm; DFCP4 differed significantly between migraine patients and healthy control subjects and was deemed a specific DFCP, while the remaining DFCPs were considered as general. Ratio distributions of WQCP samples corresponding to the migraine and normal control groups under 17 clusters were compared (Figure 3). DFCP4 showed a greater difference between the 2 groups than the other DFCPs; meanwhile, the ratio distribution was broader in migraine patients than in normal control subjects, implying that DFCP4 comprised a large number of samples from the former but few from the latter and could thus be a DFCP specific to migraine.

We evaluated the reproducibility of the data by random resampling. Ratio distributions under 4 resampling processes with a sampling rate of 50% and 4 with a sampling rate of 90% showed 1 cluster that differed significantly between the 2 groups (Supplementary Figure S1). There was a strong correlation between this cluster and DFCP4, indicating that it corresponded to the specific DFCP. Correlation coefficients are listed in Supplementary Table S2.

In order to illustrate the differences caused by different window lengths, we computed the DFCPs based on the window length of 12 s and 60 s using the Beijing_Zhang dataset. Ratio distributions for the window length of 12 s and 60 s also demonstrated that there was 1 cluster that had the most significant difference between the 2 groups (Supplementary Figure S2). Besides that, the correlation coefficient (Supplementary Table S3) was strong between this cluster and DFCP4, representing that it was compatible with the specific DFCP.

For reasons of matching gender and age, we take the ICMP dataset for the control group with the window length for 12 s, 36 s, and 60 s to extract the DFCPs. Ratio distributions for the window length of 12 s, 36 s, and 60 s manifested that there was a significant difference in 1 cluster between the 2 groups (Supplementary Figure S3), which also has a strong correlation with DFCP4 implying that it was consistent with the specific DFCP. Correlation coefficients are listed in Supplementary Table S4.

### 3.2. Specific DFCP

**Figure 4:**
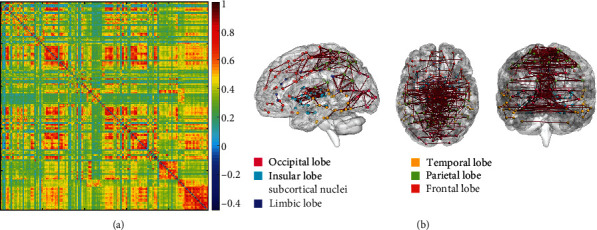
DFCPs in migraine patients. (a) DFCP4 in full matrix view. (b) DFCP4 projected onto a standard brain surface. A threshold > 0.75 was defined to retain higher connective edges.

**Figure 5:**
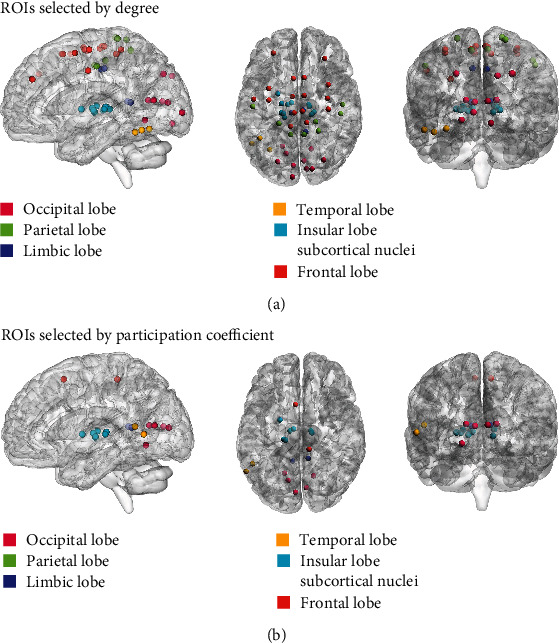
ROIs in migraine patients selected based on network metrics. (a, b) ROIs with AUC values of degree (a) and participation coefficient (b) >1 standard deviation above the mean value.

**Figure 6:**
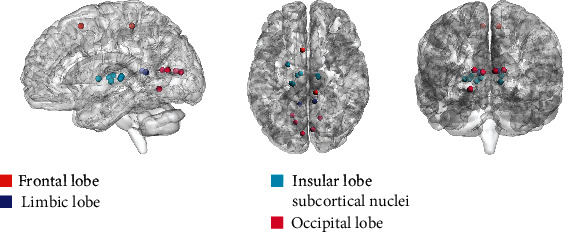
Critical ROIs projected onto a standard brain surface.

A full matrix view of DFCP4 for the migraine group is shown in Figure 4(a), and connectivity patterns with a strength > 0.75 projected onto a standard brain surface are shown in Figure 4(b) as previously described [33, 34]. The degree and participation coefficient of DFCP4 were calculated in order to identify brain areas critical for migraine. ROIs whose AUC value of degree or participation coefficient was >1 standard deviation above the mean value in DFCP4 are shown in Figure 5; the critical ROIs detected by the intersection of these 2 types of ROI are shown in Table 1.  Figure 6 shows the critical ROIs for DFCP4 projected onto a standard brain surface; this was visualized, and connectivity strength was determined, using BrainNetViewer [42].

### 3.3. General DFCPs and Intergroup Differences

**Figure 7:**
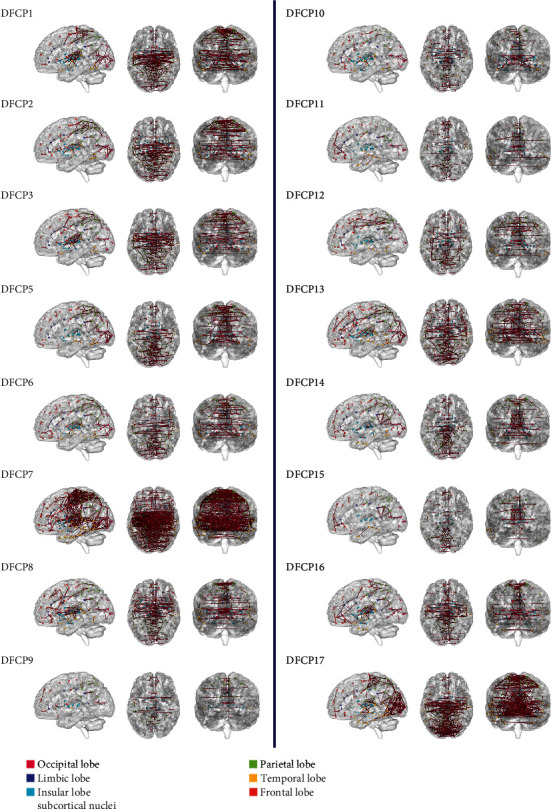
Visualization of 16 general DFCPs on a standard brain surface in migraine patients and normal control subjects. ROIs are shown as colored spheres, and FC values between ROIs represented by Pearson's correlation coefficients are shown as brown edges. A threshold > 0.75 was defined to retain higher connective edges. Each brain area is shown as a different color.

**Figure 8:**
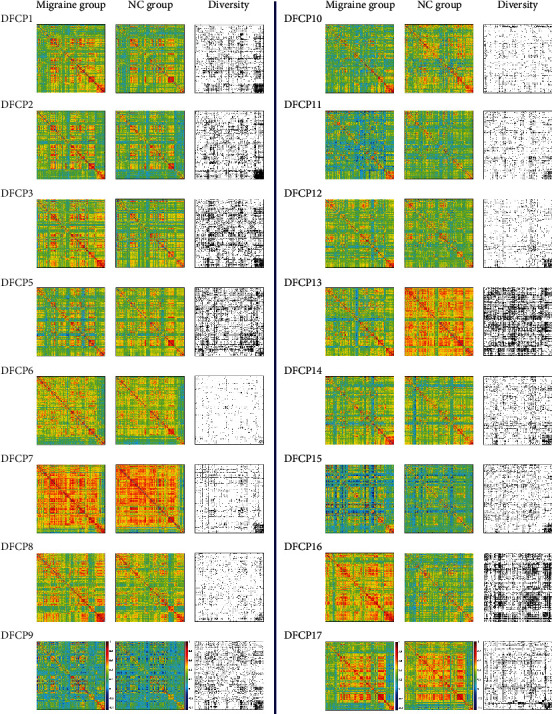
Visualization of 16 general DFCP centroids and intergroup difference matrices. Centroids with dimensions of 246 × 246 in migraine patients and normal control subjects are shown as jet colormaps, and 246 × 246 intergroup difference matrices are shown as gray colormaps. DFCP centroids are depicted in full matrix view and color-coded according to the strength of each DFC matrix. Intergroup differences are shown as a gray matrix, and a darker color represents a significantly different value.

**Figure 9:**
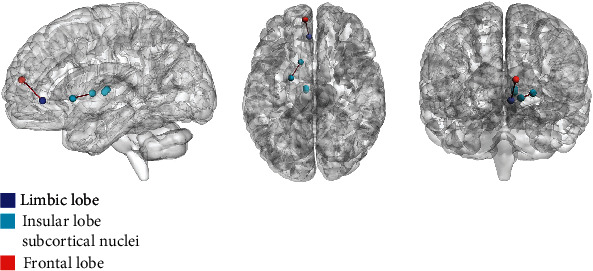
Visualization of 3 dFCs differing significantly for all general DFCPs on a standard brain surface. Colored spheres represent brain regions, and brown edges denote the connection between them.

The 16 general DFCPs were visualized and projected onto a standard brain surface (Figure 7). The strength of the edges was >0.75.  Figure 8 shows information pertaining to the 16 general DFCPs for the migraine and normal control groups. By analyzing the intersection of significantly different dFCs in each general DFCP, we identified 3 dFCs that were present in all general DFCPs; these were projected onto the cortical surface (Figure 9). And the ROIs of the 3 dFCs were mainly distributed in Brodmann area 10, Brodmann area 32, the basal ganglia, and the thalamus. Detailed information about each brain region in the 3 dFCs is presented in Table 2.

## 4. Discussion

### 4.1. DFCP Specific to Migraine

The results of this study demonstrate that migraine is associated with a specific DFCP. Several neuroimaging studies have shown that pain elicits activity across a network of brain regions (referred to as the pain matrix) that includes the primary (S1) and secondary (S2) somatosensory cortices, anterior cingulated cortex (ACC), supplementary motor cortex area, PFC, thalamus, amygdala, basal ganglia, and insula [43–45]. The sensation of pain is thought to be caused not only by activation of 1 or more specific brain regions in the pain matrix but also by information flow and integration in these areas [46, 47]. Brodmann area 10 may play an important role in the collation, integration, and high-level processing of nociceptive information and pain [48]. In addition, imaging studies have revealed abnormal activity in brain regions associated with pain information processing in migraine patients, including the ACC, thalamus, insula, and PFC [49–51]. In the present study, DFCP4 brain regions with strong connectivity included Brodmann area 1/2/3 (primary somatosensory cortex in the postcentral gyrus—i.e., S1), Brodmann area 6 (secondary motor cortex—i.e., S2), Brodmann area 10 (anterior PFC), Brodmann areas 24 and 32 (ventral and dorsal ACCs, respectively), insular gyrus, basal ganglia, and thalamus (Figure 4), indicating that these ROIs are closely related to pain processing. ROIs that are not part of the pain matrix but have also been linked to migraine or pain were Brodmann area 4 (primary motor cortex), Brodmann area 5 (superior parietal lobule), Brodmann area 7 (visuomotor coordination), posterior parietal cortex, and medioventral occipital cortex, which is consistent with findings from previous studies on migraine and pain. For instance, stimulation of the motor cortex has been used to treat chronic pain [52], and stimulation of Brodmann's area 5/7 revealed this region as being among the most closely associated with pain perception [53]. FC in the left posterior cingulate cortex (PCC) was found to be increased in migraineurs without aura [54]. The involvement of the visual cortex could explain why migraine patients experience photophobia. Thus, DFCP4 identified in this study may reflect the neurologic mechanisms of migraine.

In order to investigate this DFCP in greater detail, we analyzed network metrics. According to the degree, the selected ROIs in DFCP4 were mainly distributed in Brodmann areas 1/2/3, 4, 8, 23, and 37; occipital lobe, basal ganglia; and thalamus. According to participation coefficients, the selected ROIs in DFCP4 were mainly distributed in Brodmann areas 1/2/3 (lower limb region), 4 (lower limb region), 8 (medial region), 23 (ventral area), and 37 (dorsolateral area); caudoposterior superior temporal sulcus; occipital lobe, basal ganglia; and thalamus. From the intersection of the two sets of ROI, we identified 16 critical ROIs that were mainly distributed in Brodmann areas 1/2/3 (lower limb region), 8 (medial region), and 23 (ventral area); occipital lobe; basal ganglia; and thalamus. Brodmann areas 1/2/3 and 8, basal ganglia, and thalamus belong to the pain matrix whereas Brodmann area 23 and occipital lobe are implicated in migraine.

The functions of ROI1 include language cognition, working memory, and pain anticipation [55]; ROI66 has been linked to pain localization, movement, and swallowing; ROI181 and ROI182 are involved in cognition and emotion; and ROI191, ROI192, ROI195, ROI197, and ROI198 are related to memory, cognition, and visual perception. Besides their involvement in pain, ROI221, ROI231, ROI234, and ROI235 are associated with the imagination and execution of actions; ROI239 is implicated in the execution of actions; and ROI245 and ROI246 are involved in pain monitoring, pain perception in somesthesis, and execution of actions. Of the 16 ROIs in DFCP4, 9 were related to pain processing. Additionally, emotion, cognition, action, and vision are influenced in migraine, which could explain why migraine patients may experience vision and memory problems as well as cognition dysfunction [56, 57].

### 4.2. Similarities and Differences in General DFCPs

There were 16 general DFCPs that reflected similarities in brain function between migraine patients and normal subjects. In the resting state, we observed temporal variations in FC patterns (Figures 7 and 8), which is consistent with previous reports [31, 33, 34]. All of the general DFCPs involved several regions of the default mode network (DMN) [58, 59], which is associated with dFC [60]. The core areas in the DMN are the mPFC, PCC, superior frontal cortex, precuneus, inferior parietal lobule, lateral temporal cortex, and parahippocampal gyrus [61, 62]. In addition to having overlapping brain regions, each DFCP differed from the DMN. The ROIs of DFCP14 and DFCP15 were the most similar to the DMN, with direct connections between functional hubs such as PCC and mPFC. Moreover, these two DFCPs covered the parietal and occipital lobes, with sparser connections in DFCP15 than in DFCP14. DFCP1, DFCP2, and DFCP3 involved DMN activities but had more interhemispheric connections in the parietal lobe, insular lobe, basal ganglia, and thalamus. Besides connections in the DMN, DFCP5 DFCP6, and DFCP17 showed more interhemispheric connections in the parietal, frontal, and occipital lobes; DFCP6 involved more frontal lobe activity whereas DFCP17 had a higher density of connections, especially in the visual area. DFCP8, DFCP13, and DFCP16 also exhibited activities in the DMN but showed greater FC in the frontal and temporal lobes and subcortical nuclei, with DFCP8 and DFCP13 involving more connections in the frontal and occipital lobes, respectively. DFCP10 and DFCP3 involved activities in the DMN; the former had more interhemispheric connections in subcortical nuclei and the latter in the frontal lobe and thalamus, with more long-distance connections. DFCP12 had connections in the parietal and frontal lobes with long connections between these 2 areas. DFCP7 showed the highest degree of complexity and the largest number of connections in all brain regions, indicating that the brain is highly active in the resting state [33, 34]; this is evidenced by the fact that it consumes approximately 20% of the energy produced by the body while accounting for just 2% of total body weight [63, 64]. DFCP9 had just a few sparse connections, demonstrating that the brain was in a true resting state.

Migraine patients exhibited abnormal dFC in general DFCPs compared to normal control subjects (Figure 8). Three pairs of dFCs were present in each DFCP (Figure 9 and Table 2). The ROIs of these dFCs were distributed in A10m (Brodmann area 10 in the superior frontal lobe), A32sg (Brodmann area 32 in ACC), basal ganglia, and thalamus and are components of the pain matrix. Thus, even among general DFCPs, there are functional differences in those ROIs that may have clinical significance for migraine.

### 4.3. Static FC and DFCP Specific to Migraine

In this paper, the critical ROIs of the static network were extracted in the same way based on static functional connectivity, which was referred to as Supplementary Table S5. According to the two attributes, 15 critical ROIs can be extracted. Compared with DFCP4, ROI181, ROI182, ROI195, and ROI197 were extracted at the same time. The ROIs in the static network were mainly in the frontal lobe, limbic lobe, and occipital lobe. In addition to the above brain regions, the specific DFCP also extracted the brain regions distributed in basic ganglia and subcortical nucleus, which were also associated with migraine in previous studies. It could be seen that these were the critical ROIs revealed by changing a time scale from static to dynamic functional connectivity, which can provide different perspectives and references to understand the neural mechanism of migraine.

### 4.4. Different Dataset and Different Window Lengths

According to the results, the specific DFCPs obtained from different datasets and different window lengths have a strong correlation. This may be caused by the following reasons. First of all, this paper uses the method of segment, averaging, and clustering, which may reduce the impact of different window lengths. As no matter how long the window length is, the dFCS matrix would be automatically divided into different segments then be averaged, which might reduce the inconsistency caused by different window lengths. Secondly, when the time scale becomes dynamic and smaller, it can better reveal the fine information of network nodes that may not be revealed by the static network and might reduce the impact of different TR from different datasets. Therefore, the specific DFCP obtained by this method has certain reliability and repeatability.

### 4.5. Segmenting and Clustering

In previous studies [65, 66], dynamic functional connectivity was used to consider a single sample for the clustering approach which is an excellent approach. In our study, it could be seen from Figure 2 that adjacent dFCS are similar in color, so the illustrated dFCS matrix could be divided into several segments based on similarities in the color of adjacent rows to reduce the calculation amount and improve efficiency. For this reason, we adopt the method of segmentation in advance and then clustering. Furthermore, the approach of automatic localization can avoid the error caused by humans, which is beneficial to the wide application of this method.

### 4.6. Limitations and Future Directions

This study had some limitations. Firstly, we did not analyze the various subtypes of migraine, which may have distinct DFCPs. Secondly, the temporal relationships between DFCPs were not investigated. In future studies, we will construct a model based on the migraine-specific DFCP and associated ROIs that can serve as a diagnostic reference for clinicians and validate the specificity and sensitivity of this model.

## 5. Conclusion

In this study, we used the A-DFCM with twice-clustering to elucidate the neurologic basis of migraine patients. In this model, a novel method of automatically generating WQCPs from rs-fMRI data improved the efficiency and accuracy of segmentation, and *K*-means clustering combined with hierarchical clustering eliminated randomness. Using this approach and the independent 2-sample *t*-test, we identified 17 DFCPs including 16 that were general and 1 that was closely related to the pathologic features of migraine and could serve as a sensitive and specific neuroimaging diagnostic biomarker. Another important finding of our work was that the general DFCPs were associated with 6 critical brain areas and are thus complementary neuroimaging features that can help to distinguish migraine patients from normal subjects.

## Figures and Tables

**Table 1:** Critical regions of interest (ROIs)^∗^.

ROI number	Abbreviation	Anatomic and modified cytoarchitectonic description
1	A8m	Medial Brodmann area 8 in superior frontal gyrus of frontal lobe
66	A1/2/3ll	Brodmann area 1/2/3 (lower limb region) in paracentral lobule of frontal lobe
181	A23v	Brodmann area 23 (ventral area) in cingulate gyrus of limbic lobe
182	A23v	Brodmann area 23 (ventral area) in cingulate gyrus of limbic lobe
191	rCunG	Rostral cuneus gyrus in ventromedial occipital cortex of occipital lobe
192	rCunG	Rostral cuneus gyrus in ventromedial occipital cortex of occipital lobe
195	rLinG	Rostral lingual gyrus in ventromedial occipital cortex of occipital lobe
197	vmPOS	Ventromedial parieto-occipital sulcus in ventromedial occipital cortex of occipital lobe
198	vmPOS	Ventromedial parieto-occipital sulcus in ventromedial occipital cortex of occipital lobe
221	GP	Globus pallidus in basal ganglia
231	mPFtha	Medial prefrontal thalamus in subcortical nuclei
234	mPMtha	Premotor thalamus in subcortical nuclei
235	mPMtha	Premotor thalamus in subcortical nuclei
239	PPtha	Posterior parietal thalamus in subcortical nuclei
245	rLinG	Lateral prefrontal thalamus in subcortical nuclei
246	lPFtha	Lateral prefrontal thalamus in subcortical nuclei

**Table 2:** Regions of interest (ROI)^∗^ in the 3 dynamic functional connectivity.

ROI number	Abbreviation	Anatomic and modified cytoarchitectonic description
13	A10m	Medial Brodmann area 10 in superior frontal gyrus of frontal lobe
187	A32sg	Subgenual Brodmann area 32 in cingulate gyrus of limbic lobe
219	vCa	Ventral caudate in basal ganglia of subcortical nuclei
221	GP	Globus pallidus in basal ganglia of subcortical nuclei
231	mPFtha	Medial prefrontal thalamus in thalamus of subcortical nuclei
237	rTtha	Rostral temporal thalamus in thalamus of subcortical nuclei

^∗^ROI descriptions and abbreviations were obtained from the Brainnetome Atlas [30].

## Data Availability

The data of migraineurs were obtained from the Department of Neurology of Shanghai Sixth People's Hospital East Affiliated to Shanghai University of Medicine & Health Science and were approved by the Independent Ethics Committee of Shanghai Sixth People's Hospital East Campus. According to the Regulations on Human Genetic Resources Management published by the Chinese government and in order to ensure the privacy of patients, these data are not available. The data of normal controls were obtained from a free public database which can be accessed at http://fcon_1000.projects.nitrc.org/fcpClassic/FcpTable.html.

## Supplementary Materials

Supplementary Materials The online version of this article contains supplementary material, which includes the supporting tables and figures for this paper and is available to authorized users.
