# Supplementary material for: Extraction and Analysis of Dynamic Functional Connectome Patterns in Migraine Sufferers: A Resting-State fMRI Study
Source: Comput Math Methods Med. 2021 Apr 17;2021:6614520. doi: 10.1155/2021/6614520 (PMC8075661; doi:10.1155/2021/6614520)
Supplement: Supplementary Materials — The online version of this article contains supplementary material, which includes the supporting tables and figures for this paper and is available to authorized users. [file 6614520.f1.docx]

Supplementary Material

# Supplementary Data

## Supplementary Tables

Table S1 | Network metrics used in this study

| **Network metric** | **Description** | **Definition** | **Explanation** |
| --- | --- | --- | --- |
| Degree | Number of links connected to a node | $K_{i}=\sum_{j\epsilon N} a_{ij}$ | N is the set of all nodes in the network; (i, j) is a link between nodes i and j, (i, j $\epsilon$ N); and $a_{ij}$ is the status of the connection between i and j. |
| Participation coefficient | Measure of how  well-distributed links of a node are among different modules | $P_{i}=1- \sum_{s=1}^{N_{M}} {(\frac{k_{is}}{K_{i}})}^{2}$ | $N_{M}$is the number of modules; $k_{is}$is the number of links of node i to nodes in module s; and $K_{i}$ is the total degree of node i. |

Table S2 | Correlation coefficients between selected clusters and DFCP4

| **Sampling rate** | **Number** | **Cluster** | **Correlation coefficient** |
| --- | --- | --- | --- |
| 50% | 1 | 7 | 0.9265 |
| 50% | 2 | 11 | 0.9480 |
| 50% | 3 | 15 | 0.9492 |
| 50% | 4 | 15 | 0.9321 |
| 90% | 1 | 4 | 0.9391 |
| 90% | 2 | 15 | 0.9773 |
| 90% | 3 | 10 | 0.9709 |
| 90% | 4 | 10 | 0.9813 |

Table S3 | Correlation coefficients between selected clusters and DFCP4 with different window lengths

| **Widow length** | **Cluster** | **Correlation coefficient** |
| --- | --- | --- |
| 12s | 11 | 0.8399 |
| 36s | 4 | 1 |
| 60s | 6 | 0.9862 |

Table S4 | Correlation coefficients between selected clusters and DFCP4 with different window lengths for ICMP dataset

| **Widow length** | **Cluster** | **Correlation coefficient** |
| --- | --- | --- |
| 12s | 5 | 0.8432 |
| 36s | 12 | 0.9605 |
| 60s | 8 | 0.9746 |

Table S5 | Critical regions of interest (ROIs)* extracted from static FC

| **ROI number** | **Abbreviation** | **Anatomic and modified cytoarchitectonic description** |
| --- | --- | --- |
| 105 | A37mv | Brodmann area 37 (medioventral area) in fusiform gyrus of frontal lobe |
| 107 | A37lv | Brodmann area 37 (lateroventral area) in fusiform gyrus of frontal lobe |
| 108 | A37lv | Brodmann area 37 (lateroventral area) in fusiform gyrus of frontal lobe |
| 150 | A5m | Brodmann area 5 (medial area (PEm)) in precuneus of parietal lobe |
| 181 | A23v | Brodmann area 23 (ventral area) in cingulate gyrus of limbic lobe |
| 182 | A23v | Brodmann area 23 (ventral area) in cingulate gyrus of limbic lobe |
| 185 | A23c | Brodmann area 23 (caudal area 23) in cingulate gyrus of limbic lobe |
| 186 | A23c | Brodmann area 23 (caudal area 23) in cingulate gyrus of limbic lobe |
| 189 | cLinG | Caudal lingual gyrus in occipital lobe |
| 195 | rLinG | Rostral lingual gyrus in ventromedial occipital cortex of occipital lobe |
| 196 | rLinG | Rostral lingual gyrus in ventromedial occipital cortex of occipital lobe |
| 197 | vmPOS | Ventromedial parieto-occipital sulcus in ventromedial occipital cortex of occipital lobe |
| 198 | vmPOS | Ventromedial parieto-occipital sulcus in ventromedial occipital cortex of occipital lobe |
| 202 | V5/MT+ | area V5/MT+ in lateral occipital cortex of occipital lobe |
| 209 | lsOccG | lateral superior occipital gyrus in lateral occipital cortex of occipital lobe |

## Supplementary Figure


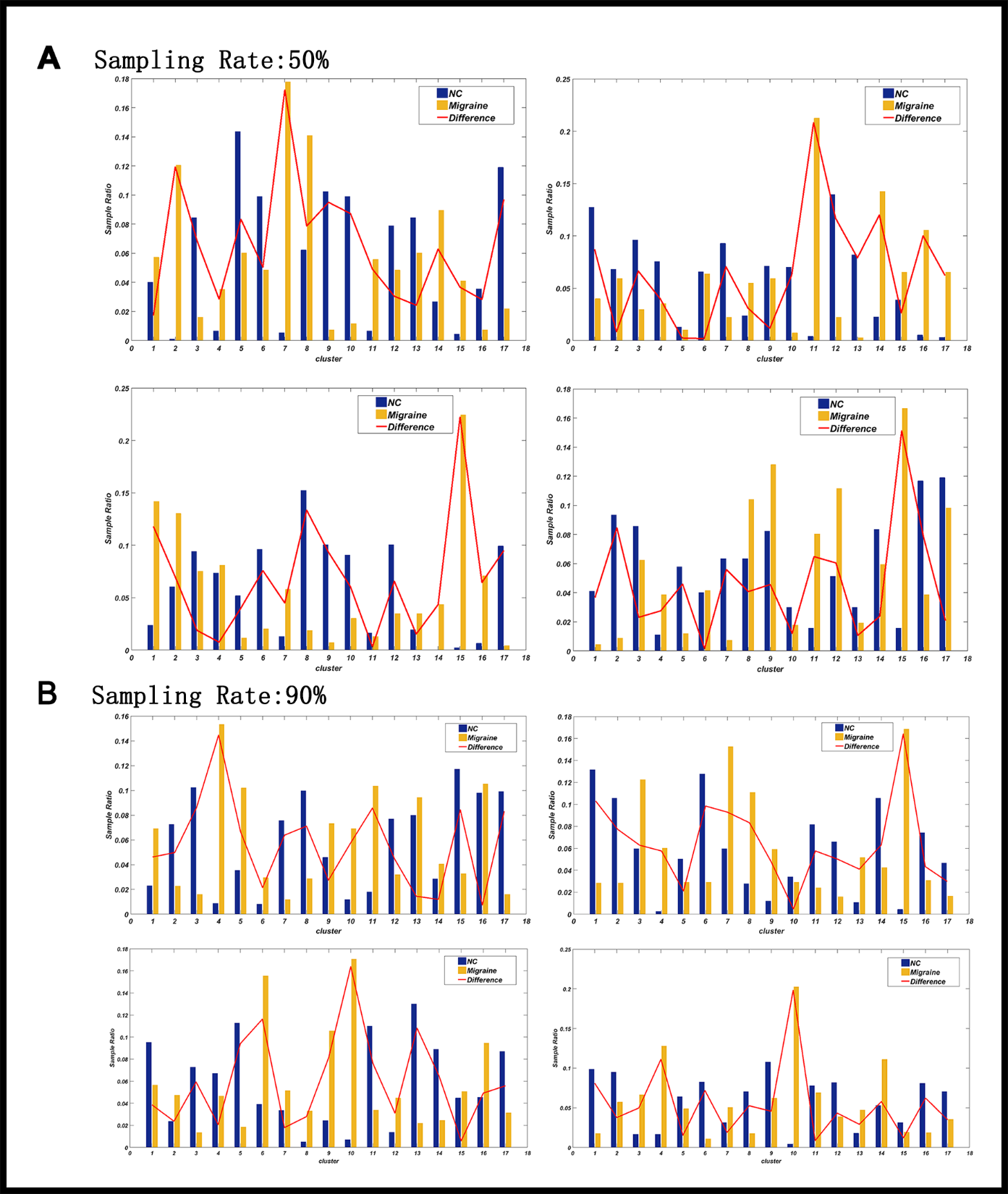


**Figure S1 | Ratio distributions of WQCP samples for migraine patients and normal control subjects under 8 resampling processes.** (A, B) Ratio distributions with sampling rates of 50% (A) and 90% (B).

**
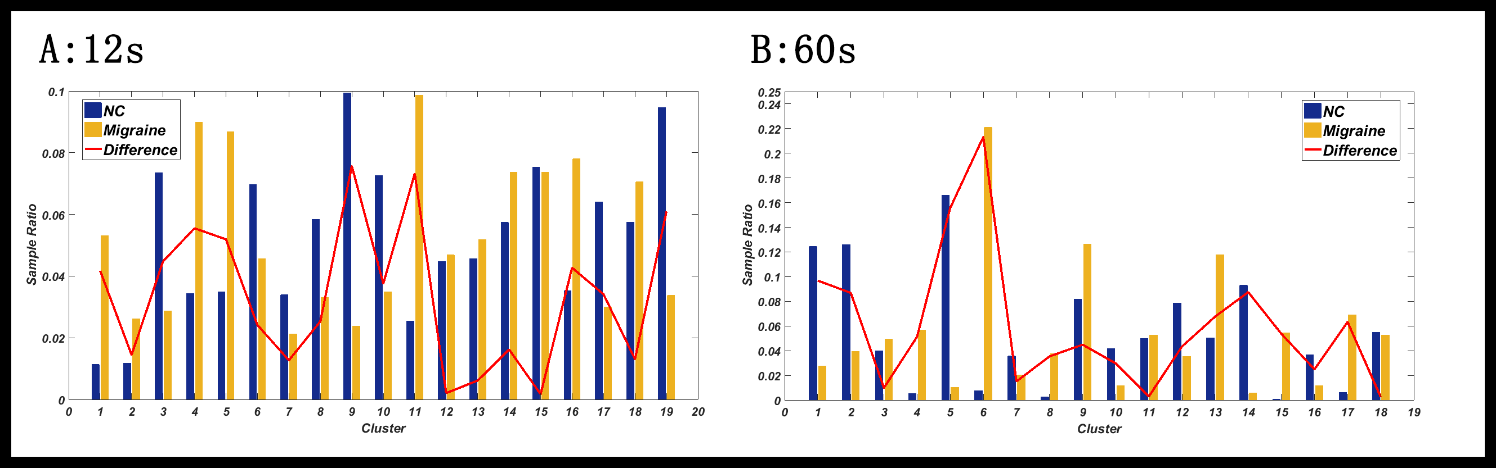
**

**Figure S2 | Ratio distributions of WQCP samples for migraine patients and normal control subjects (Beijing_Zhang dataset) under different window lengths (12s and 60s).**


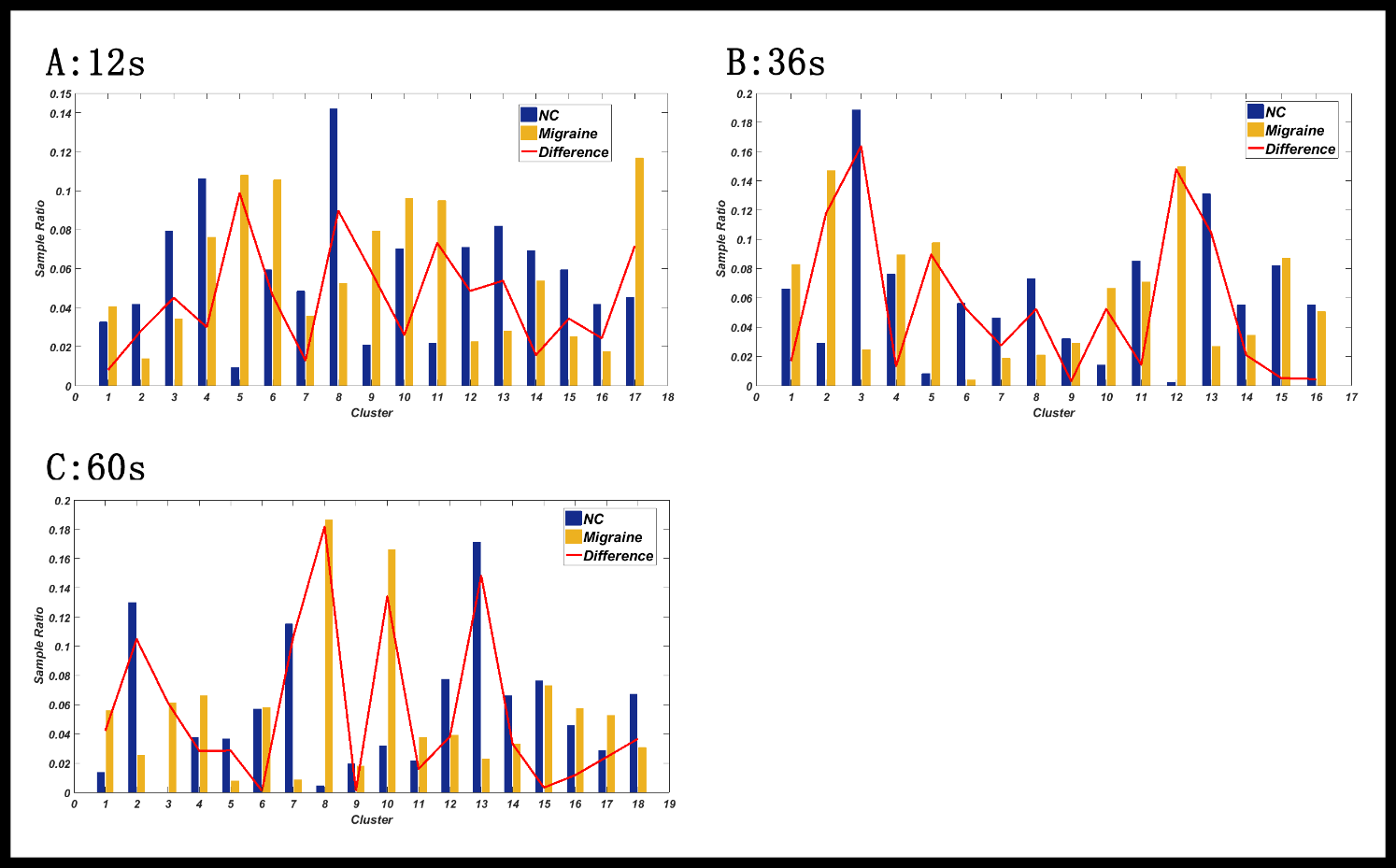


**Figure S3 | Ratio distributions of WQCP samples for migraine patients and normal control subjects(ICMP dataset) under different window lengths (12s, 36s, and 60s).**
